# Prognostic relevance of short-term changes in body weight, renal indices, and echocardiographic variables after intravenous diuretic therapy in dogs with myxomatous mitral valve disease hospitalized for pulmonary edema

**DOI:** 10.1080/01652176.2026.2648250

**Published:** 2026-03-24

**Authors:** Sin-Wook Park, Kyung-Ho You, Keon Kim, Woong-Bin Ro, Chang-Min Lee

**Affiliations:** a Department of Veterinary Internal Medicine, College of Veterinary Medicine and BK21 FOUR program, Chonnam National University, Gwangju, Korea; b Sky animal medical center, Gwangju, Korea; c The Care animal medical center, Guri, Gyeonggi-do, Korea; d Laboratory of Veterinary Internal Medicine, College of Veterinary Medicine and Veterinary Medical Research Institute, Jeju National University, Jeju, Republic of Korea

**Keywords:** Canine, cardiogenic pulmonary edema, congestive heart failure, decongestion, echocardiography, intravenous diuretic therapy, myxomatous mitral valve disease, prognosis, survival analysis

## Abstract

Congestive heart failure (CHF) secondary to myxomatous mitral valve disease (MMVD) is managed with diuretics, yet the prognostic value of short-term hemodynamic responses remains unclear. This retrospective study evaluated 50 dogs with ACVIM stage C or D MMVD hospitalized for CHF. We assessed changes in body weight, echocardiographic parameters, and renal values before and after intravenous (IV) diuretic therapy to determine their association with survival. IV diuretic therapy led to significant reductions in body weight and echocardiographic indices, including the left atrium-to-aortic root ratio (LA/Ao), normalized left ventricular internal diameter in diastole (LVIDDN), and early diastolic transmitral flow velocity (all *P *< 0.005). Conversely, blood urea nitrogen and creatinine concentrations significantly increased (*P* < 0.001). However, neither short-term changes in these variables nor post-treatment values were associated with long-term survival. Baseline LA/Ao and LVIDDN were related to survival in univariable analysis, while prior oral diuretic therapy was the only independent predictor in the multivariable model (Hazard Ratio 4.21; *P* < 0.001). Dogs with prior diuretic use had shorter median survival (194 days) than those without (459 days). Thus, short-term echocardiographic improvements reflect effective decongestion rather than long-term prognosis. Prognostic evaluation should prioritize baseline assessments and treatment history.

## Introduction

Myxomatous mitral valve disease (MMVD) is the most common acquired cardiac disorder in small-breed dogs and is a major cause of morbidity and mortality (Boswood et al. 2015). The disease is characterised by progressive myxomatous degeneration of the mitral valve leaflets, resulting in chronic mitral regurgitation and compensatory remodelling of the left atrium and ventricle (Borgarelli and Haggstrom 2010; Keene et al. 2019). As the condition advances, approximately one-third of affected dogs develop congestive heart failure (CHF), which substantially worsens clinical outcomes (Borgarelli and Haggstrom 2010; Keene et al. 2019).

Numerous clinical and echocardiographic variables have been evaluated as prognostic indicators in dogs with MMVD (Borgarelli et al. 2008; Sargent et al. 2015; Baron Toaldo et al. 2018, 2021; Tidholm and Häggström 2022; Vezzosi et al. 2025). Echocardiographic indices such as the left atrium-to-aortic root ratio (LA/Ao) and normalised left ventricular internal diameter in diastole (LVIDDN) reflect the degree of cardiac remodelling and have been consistently associated with disease severity and survival (Borgarelli et al. 2008; Nakamura et al. 2014). Composite systems such as the Mitral INsuffficiency Echocardiographic score have also been proposed to integrate structural and functional data for improved risk assessment (Vezzosi et al. 2021). However, these indicators mainly represent static disease status and do not account for short-term hemodynamic changes that may occur during treatment.

Intravenous diuretics, particularly furosemide, are the cornerstone of decongestive therapy for dogs hospitalised with acute CHF secondary to MMVD and generally produce rapid clinical improvement (Keene et al. 2019; Giorgi et al. 2022; Sabetti et al. 2025). However, the degree of response to diuretic therapy differs among individuals, and its prognostic significance remains uncertain. In human patients with acute heart failure, several studies have explored the association between short-term echocardiographic changes during hospitalisation and long-term clinical outcomes (Ramasubbu et al. 2012; Deferm et al. 2020; Barki et al. 2024); however, such investigations remain limited in veterinary medicine.

We hypothesised that short-term improvements in echocardiographic and clinical parameters following intravenous diuretic therapy would reflect effective decongestion and could be associated with better long-term outcomes. Therefore, this retrospective study investigated whether such short-term changes were related to survival in dogs with CHF secondary to MMVD. We aimed to evaluate whether short-term changes in body weight, renal indices, and echocardiographic variables following intravenous diuretic therapy are associated with long-term survival in dogs hospitalised for CHF secondary to MMVD, and to explore the influence of prior oral diuretic exposure on clinical status and outcomes.

## Materials and methods

### Animals

Medical records from The Care Animal Medical Centre collected between October 2021 and December 2024 were retrospectively reviewed. Dogs were eligible for inclusion if they had MMVD classified as American College of Veterinary Internal Medicine (ACVIM) stage C or D disease and were hospitalised for CHF requiring intravenous diuretic therapy. Staging followed ACVIM criteria; stage D was defined as refractory CHF requiring ≥8 mg/kg/day furosemide-equivalent diuretics (Keene et al. 2019). To be included, patients were required to have echocardiographic examination, body weight measurement, and biochemistry (plasma urea nitrogen [BUN] and creatinine) analysis performed at the time of admission, immediately prior to the initiation of intravenous diuretic therapy (baseline). Paired data were obtained during the same hospitalisation after clinical stabilisation had been achieved, defined primarily by a resting or sleeping respiratory rate ≤30 breaths/min with improvement in respiratory effort at rest. Thoracic radiographs demonstrating partial or complete resolution of CHF-consistent pulmonary infiltrates were used as supportive evidence of decongestion, particularly when serial resting/sleeping respiratory rate measurements were unavailable or inconsistently documented (e.g. due to patient anxiety/excitement limiting acquisition of resting values).

Dogs were categorised into either the first-episode group, which included those without prior cardiac medication, or the recurrent group, which included dogs that had previously received oral diuretics for recurrent or chronic CHF consistent with advanced stage C or D disease. For dogs with multiple hospitalisations, only data from the first admission that met the inclusion criteria were analysed to avoid duplication. The exclusion criteria were as follows: congenital cardiac abnormalities or other acquired cardiac diseases (e.g. bacterial endocarditis, dilated cardiomyopathy, pulmonary stenosis).

### Echocardiographic data

All echocardiographic examinations were performed by two board-certified veterinary radiologists and one board-certified veterinary internist and were subsequently reviewed by another board-certified veterinary internist. The value recorded for each measurement consisted of the average of three cardiac cycles. The LA/Ao were obtained from the right parasternal short-axis two-dimensional (2D) view as previously described (Hansson et al. 2002), and the left ventricular internal diameter at end-diastole (LVIDD) was obtained from the right parasternal short-axis 2D view (Cornell et al. 2004). Normalised dimensions were calculated according to the following formula: LVIDDN = LVIDd(cm)/(BW (kg))^0.294^ (Cornell et al. 2004; Keene et al. 2019). The peak velocity of the early diastolic transmitral flow (E) was obtained from a left apical 4-chamber view (Schober et al. 2008).

### Statistical analysis

All statistical data were analysed using commercial software (IBM SPSS Statistics, version 27, IBM Co., United States). Data were tested for normality using the Shapiro-Wilk test. Descriptive statistics are reported as the mean ± standard deviation (SD) for normally distributed continuous variables and as the median (interquartile range, IQR) for non-normally distributed continuous variables. Paired *t*-tests were used to compare the pre- and post-treatment values of the LVIDD, LVIDDN, LA/Ao, and E peak, whereas the Wilcoxon signed-rank test was applied for body weight, BUN, and creatinine. For comparisons between the first-episode and recurrent groups, normally distributed continuous variables were analysed using independent samples t-tests, whereas non-normally distributed variables were compared using the Mann–Whitney U test.

Follow-up information was obtained from recheck visits documented in the medical records and, when necessary, by telephone contact with owners and/or referring veterinarians. Owing to the retrospective design, the timing and frequency of follow-up evaluations were not standardised and were determined by clinician preference and owner compliance. The study end-point (date and cause of death/euthanasia) was established based on the medical record and/or owner/referring veterinarian report and was confirmed by telephone contact with the owner when feasible. Dogs were censored at the date of last follow-up if they were alive at the study conclusion or lost to follow-up. Cardiac-related death (including euthanasia for cardiac reasons) was considered the event. Univariable Cox proportional hazards analyses were conducted to identify potential predictors of survival using variables obtained before and after diuretic therapy, and their percentage changes (Δ%) as well as hazard ratios (HRs) with 95% confidence intervals (CIs) were calculated. Variables with *P* < 0.05 in the univariable analyses were subsequently entered into separate multivariable Cox proportional hazards models for the pretreatment data. Kaplan–Meier survival curves with log-rank tests were employed to compare the median survival times (MSTs) between groups. Survival time was calculated from the first day of hospitalisation that met the inclusion criteria to the date of death or censoring. Statistical significance was set at *P* < 0.05.

## Results

### Characteristics

A total of 171 dogs with MMVD were diagnosed with CHF during the study period; of these, 50 met the inclusion criteria. Among the 121 excluded dogs, 10 were managed as outpatients, and 111 had received intravenous diuretics before baseline echocardiography and thus did not meet the inclusion criteria. Forty-nine dogs were classified as having ACVIM stage C MMVD, and one was classified as having stage D MMVD. Of these, 31 dogs presented with their first episode of CHF, while 19 had a history of prior oral diuretic therapy and were subsequently classified into the recurrent group. The most frequently represented breeds were Maltese (*n* = 18), mixed breed (Sabetti et al. 2025), Poodle (Vezzosi et al. 2021), Shih Tzu (Vezzosi et al. 2021), Pomeranian (Vezzosi et al. 2025), Chihuahua (Keene et al. 2019), and Yorkshire Terrier (Borgarelli and Haggstrom 2010). Other breeds included Miniature Schnauzer (*n* = 1) and Cocker Spaniel (Boswood et al. 2015). Overall, 31 dogs were male, of which 28 were neutered, and 19 were female, of which 15 were spayed. At baseline, the mean age was 11.5 ± 2.5 years, the mean heart rate was 155 ± 26 beats per minute, and the median systolic blood pressure was 132 mmHg (IQR, 120–150). Extra-cardiac comorbidities at baseline were documented in 30/50 (60%) dogs and are summarised in Table S1. The most common comorbidities were urogenital/urinary/reproductive (19/50, 38%; including chronic kidney disease in 13/50, 26%), followed by hepatobiliary/hepatic/splenic (13/50, 26%) and gastrointestinal (11/50, 22%) conditions.

### Comparison between the first-episode and recurrent groups

Comparative findings between the two groups are summarised in Table 1. At baseline, dogs in the recurrent group had significantly higher plasma BUN (*P* = 0.004) and creatinine concentrations (*P* = 0.008) than those in the first-episode group, whereas the patient age, sex distribution, physical examination findings (including heart rate, blood pressure, and body weight), and echocardiographic parameters were comparable between groups. Following diuretic therapy, the recurrent group showed a higher post-treatment LA/Ao (*P* = 0.009). During diuretic therapy, elevations in BUN and creatinine were greater in the first-episode group (*P* = 0.027 vs. 0.033), whereas reductions in body weight and echocardiographic parameters were comparable between groups.

**Table 1.** Comparison of clinical, renal, and echocardiographic variables between the first-episode group (*n* = 31) and recurrent group (*n* = 19).

Variable	First-episode group (*n* = 31)	Recurrent group (*n* = 19)	*P*-value
Age (years)	11.8 (10.2–13.8)	11.8 (10.2–13.1)	0.441
Sex	(21/10)	(10/9)	0.285
Hospitalisation period (day)	3 (2–4)	4 (2.5–6)	0.072
Heart rate^1^ (beat per minute)	150 ± 22	165 ± 31	0.099
Blood pressure^1^ (mmHg)	135 (124–162)	127 (120–149)	0.492
Body weight^1^ (kg)	4.00 (3.60–5.75)	4.10 (3.31–4.65)	0.549
BUN^1^ (mg/dL)	22 (17–30)	33 (25–48)	0.004
Creatinine^1^ (mg/dL)	0.9 ± 0.4	1.3 ± 0.6	0.008
LVIDDN^1^	2.01 ± 0.21	2.18 ± 0.25	0.335
LA/Ao^1^	2.19 ± 0.38	2.40 ± 0.48	0.105
E peak^1^ (cm/s)	157 (142–169)	160 (145–170)	0.795
Body weight^2^ (kg)	3.88 (3.40–5.19)	3.95 (2.91–4.49)	0.379
BUN^2^ (mg/dL)	40 (31–52)	45 (32–57)	0.644
Creatinine^2^ (mg/dL)	1.4 (1.1–1.7)	1.4 (1.2–2)	0.359
LVIDDN^2^	1.91 (1.77–2.03)	2.10 (1.96–2.23)	0.529
LA/Ao^2^	1.78 (1.70–2.00)	1.96 (1.77−2.28)	0.009
E peak^2^ (cm/s)	134 ± 28	136 ± 25	0.998
Δ Body weight (%)	–5.2 (–7.9 to –3.0)	–5.6 (–8.7 to –3.2)	0.742
Δ BUN (%)	90.0 (14.8–174.5)	24.2 (–11.7 to 92.5)	0.027
Δ CREA (%)	68.9 (23.3–131.3)	16.7 (–3.3 to 67.5)	0.033
Δ LVIDD (%)	–5.4 ± 9.7	–6.0 ± 8.3	0.835
Δ LVIDDN (%)	–3.9 ± 9.8	–4.0 ± 8.2	0.980
Δ LA/Ao (%)	–16.1 ± 13.2	–8.5 ± 15.5	0.073
Δ E peak (%)	–17.7 (−26.3 to 8.0)	–12.9 (−25.7 to -4.0)	0.944

^1^ Variables measured before diuretic administration.

^2^ Variables measured after diuretic administration.

BUN, blood urea nitrogen; E peak, peak velocity of E wave of transmitral flow; LA/Ao, left atrium-to-aortic root ratio; LVIDD, left ventricular internal diameter in diastole; LVIDDN, normalised left ventricular end-diastolic internal diameter indexed to body weight; MMVD, myxomatous mitral valve disease.

### Short-term response to diuretic therapy

The median duration of hospitalisation was three days (IQR, 2–4.75), with no statistically significant differences between the first-episode and recurrent groups (*P* = 0.072). The median interval between the initial and post-stabilisation examination was two days (IQR, 1.25–4). Respiratory rate decreased from a median of 72 breaths/min (IQR, 60–78), recorded as the highest value at presentation, to a median of 18 breaths/min (IQR, 18–24), recorded as the lowest resting/sleeping respiratory rate on the day of the post-stabilisation evaluation prior to discharge. The changes related to body weight, renal variables, and echocardiographic parameters before and after treatment are summarised in Table 2. After diuretic therapy, plasma BUN and creatinine levels increased significantly (*P* < 0.001), whereas body weight and all echocardiographic parameters (LVIDD, LVIDDN, LA/Ao, and E peak) decreased compared with baseline values (*P* < 0.01).

**Table 2.** Changes in of clinical, renal, and echocardiographic variables before and after intravenous diuretic administration in 50 dogs with CHF.

Variable	Pretreatment	Post-treatment	*Δ* (%)	*P*-value
Body weight (kg)	4.09 (3.36–5.07)	3.92 (3.08–4.84)	–5.3 (–8.1 to –3.0)	<0.001
BUN (mg/dL)	26 (18–36)	41 (30–56)	65 (0–143)	<0.001
Creatinine (mg/dL)	0.9 (0.7–1.3)	1.4 (1.1–1.9)	50 (7.7–114)	<0.001
LVIDD (mm)	32.1 ± 5.6	30.3 ± 5.8	–5.7 ± 9.1	<0.001
LVIDDN	2.04 (1.9–2.24)	1.97 (1.83–2.18)	–3.9 ± 9.2	0.003
LA/Ao	2.21 (1.97–2.51)	1.89 (1.71–2.12)	–13.1 ± 14.4	<0.001
E peak (cm/s)	157 ± 23	135 ± 27	–16.1 (–26.4 to –3.2)	<0.001

Values are presented as the median (interquartile range) or mean ± standard deviation. The symbol Δ (%) represents the percentage change from pretreatment to post-treatment. All variables showed significant differences between the pretreatment and post-treatment evaluations (P<0.05). BUN, blood urea nitrogen; CHF, congestive heart failure; E peak, transmitral E wave peak velocity; LA/Ao, left atrium-to-aortic root ratio; LVIDD, left ventricular internal diameter in diastole; LVIDDN, normalised LVIDD.

### Treatments

Nineteen dogs were prescribed oral cardiac medications before presentation. All dogs received pimobendan at a median (range) dose of 0.3 mg/kg BID (0.3–0.4) and a loop diuretic, either furosemide (*n* = 8; 2 mg/kg BID (Borgarelli and Haggstrom 2010; Boswood et al. 2015; Keene et al. 2019) or torsemide (*n* = 11; 0.2 mg/kg BID [0.05–0.45]). Additional cardiac medications included spironolactone (*n* = 14; 1.0 mg/kg SID [0.5–1.5]), isosorbide dinitrate (*n* = 6; 1.0 mg/kg BID [0.5–1.5]), sildenafil (*n* = 5; 1.0 mg/kg BID [0.5–1.5]), and enalapril (*n* = 4; 0.5 mg/kg BID [0.5–1.0]). During hospitalisation, all dogs received continuous intravenous furosemide therapy, initially at 0.66–1.0 mg/kg/h, followed by intermittent intravenous bolus administration. During hospitalisation, all dogs received supplemental oxygen via an oxygen cage (FiO₂ approximately 60%) and pimobendan (0.3–0.45 mg/kg BID); the median duration of oxygen supplementation was 2 days (IQR, 1–2.25). At discharge, all dogs were prescribed pimobendan at a median (range) dose of 0.3 mg/kg BID (0.3–0.45) and a loop diuretic, either furosemide (*n* = 14; 2 mg/kg BID [1.1–2.2]) or torsemide (*n* = 36; 0.2 mg/kg BID [0.1–0.5]). Lower discharge loop diuretic doses were prescribed in selected dogs with marked azotemia/advanced chronic kidney disease as a renal-sparing approach to maintain decongestion while minimising further deterioration in renal function. Additional cardiac medications included spironolactone (*n* = 39; 1.0 mg/kg SID [0.5–2]), isosorbide dinitrate (*n* = 26; 1.0 mg/kg BID [0.5–1.5]), sildenafil (*n* = 11; 1.0 mg/kg BID [0.5–2]), and enalapril (*n* = 10; 0.5 mg/kg BID [0.25–1.5]). During hospitalisation, one dog had asymptomatic ventricular premature complexes and did not receive antiarrhythmic therapy. Atrial fibrillation was diagnosed during follow-up in one dog and was managed with diltiazem for ventricular rate control. The discharge medication combinations are summarised in Table 3.

**Table 3.** Discharge medical treatments administered to dogs hospitalised for congestive heart failure secondary to myxomatous mitral valve disease.

Medical treatments	Number of dogs
P + F	1
P + F + E	1
P + F + S	4
P + F + S + ISDN	5
P + F + S + SIL	1
P + F + E + S + SIL	1
P + F + E + S + SIL + ISDN	1
P + T	2
P + T + E	4
P + T + S	8
P + T + E + ISDN	2
P + T + S + ISDN	11
P + T + S + SIL	2
P + T + SIL + ISDN	1
P + T + E + S + ISDN	1
P + T + S + SIL + ISDN	5

E, Enalapril; F, furosemide; ISDN, isosorbide dinitrate; P, pimobendan; S, spironolactone; SIL, sildenafil; T, torsemide.

### Survival analysis

**Figure 1.**
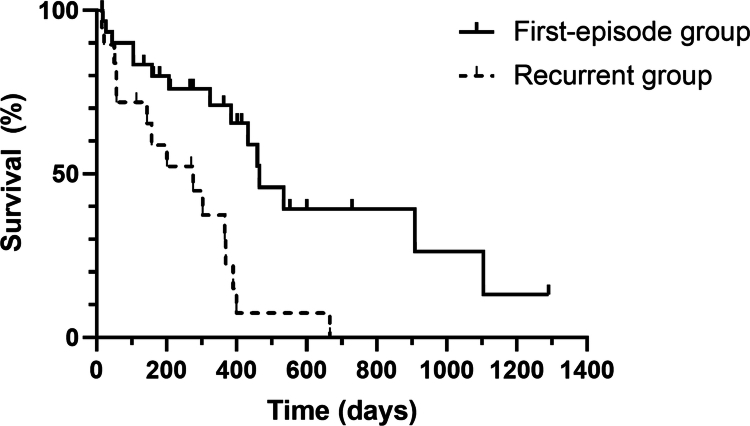
Kaplan–Meier survival curves comparing survival times according to prior oral diuretic therapy before hospitalisation in dogs with MMVD hospitalised for cardiogenic heart failure. Survival time was calculated from the date of study inclusion (index hospitalisation) to the endpoint. Dogs that had received oral diuretics before hospitalisation (recurrent group) showed significantly shorter survival than those without prior diuretic treatment (first-episode group) (median survival time, 194 vs 459 days; *P* < 0.001). Survival distributions were estimated using the Kaplan–Meier method and compared with the log-rank test. The endpoint was cardiac-related death; tick marks indicate censored observations (alive at last follow-up, lost to follow-up, or non-cardiac/undetermined death).

Of the 50 dogs included in the study, 30 (60%) died of cardiac-related causes, 13 (26%) were lost to follow-up, and 7 (14%) were still alive at the end of the observation period. The results of the univariable Cox proportional hazards analysis are shown in Table 4. Among the pretreatment variables, pre-admission oral diuretic therapy (*P* < 0.001), LVIDDN (*P* = 0.012), and LA/Ao (*P* = 0.041) met the inclusion threshold for inclusion in the multivariable analysis. In the multivariable model, only prior diuretic use was strongly associated with outcome (HR = 4.21, 95% CI 1.89–9.39, *P* < 0.001). None of the post-treatment or percentage change (Δ%) variables were significantly associated with survival (*P* > 0.05). The MST for the overall population, calculated from the date of study inclusion (index hospitalisation) to death or last follow-up, was 384 days (95% CI 343–425 days). Dogs in the first-episode group (no prior cardiac medication) had a markedly longer MST than those with prior oral diuretic therapy (459 days [95% CI 399–519] vs 194 days [95% CI 0–236]; log-rank *P* < 0.001; Figure 1).

**Table 4.** Univariate Cox proportional hazards regression associated with outcome in dogs with MMVD (*n* = 50).

Variable	Hazard ratio	95% confidence interval		*P*-value
Age (years)	1.050	0.899	1.227	0.537
Sex	0.888	0.421	1.874	0.755
Pre-admission oral diuretic therapy	4.356	1.979	9.595	<0.001
BUN^1^ (mg/dL)	1.020	0.998	1.042	0.080
Creatinine^1^ (mg/dL)	1.795	0.895	3.599	0.097
LVIDDN^1^	8.577	1.615	45.541	0.012
LA/Ao^1^	2.415	1.037	5.628	0.041
E peak^1^ (cm/s)	0.996	0.982	1.009	0.548
BUN^2^ (mg/dL)	1.001	0.987	1.016	0.869
Creatinine^2^ (mg/dL)	1.074	0.958	1.204	0.201
LVIDDN^2^	3.877	0.844	17.816	0.082
LA/Ao^2^	1.738	0.785	3.848	0.173
E peak^2^ (cm/s)	1.001	0.987	1.015	0.914
Δ Body weight (%)	0.943	0.855	1.039	0.238
Δ BUN (%)	0.998	0.995	1.002	0.272
Δ CREA (%)	1.000	0.999	1.001	0.680
Δ LVIDD (%)	0.895	0.615	1.302	0.562
Δ LVIDDN (%)	0.992	0.955	1.030	0.670
Δ LA/Ao (%)	1.007	0.975	1.041	0.667
Δ E peak (%)	1.002	0.988	1.017	0.755

^1^ Variables measured before diuretic administration.

^2^ Variables measured after diuretic administration. BUN, blood urea nitrogen; E peak, peak velocity of E wave of transmitral flow; LA/Ao, left atrium-to-aortic root ratio; LVIDD, left ventricular internal diameter in diastole; LVIDDN, normalised left ventricular end-diastolic internal diameter indexed to body weight; MMVD, myxomatous mitral valve disease.

## Discussion

This study evaluated short-term changes in body weight, biochemical values, and echocardiographic parameters following intravenous diuretic therapy in dogs hospitalised for CHF secondary to MMVD and investigated their association with survival. Although diuretic therapy led to significant reductions in body weight and cardiac dimensions that were accompanied by transient increases in BUN and creatinine, none of these short-term variations were associated with long-term outcome. These findings suggest that short-term hemodynamic improvements after decongestive therapy do not necessarily predict long-term survival, underscoring the limited prognostic value of acute treatment responses. In the survival analysis, echocardiographic indicators of cardiac remodelling before intensive treatment, including the LA/Ao and LVIDDN, were significantly associated with prognosis in univariable analysis. However, when prior oral diuretic therapy was included in the multivariable model, this factor emerged as the only variable independently associated with survival. These results suggest that in dogs with CHF secondary to MMVD, prognostic evaluation should incorporate not only echocardiographic and biochemical parameters but also information on prior diuretic use and dosage, as treatment history substantially influences long-term outcome.

Previous experimental and physiological studies have characterised the acute hemodynamic effects of furosemide and volume depletion in dogs. Fine et al. (2010) demonstrated that short-term intravascular fluid loss, whether induced by furosemide administration or water deprivation, leads to measurable reductions in left ventricular end-diastolic volume, stroke volume, and transmitral E wave velocity, consistent with decreased preload (Fine et al. 2010). Similarly, Suzuki et al. (2011) reported a dose-dependent decrease in left atrial pressure within a few hours after furosemide treatment in dogs with experimentally induced mitral regurgitation (Ishikawa et al. 2011). Both studies confirmed the strong, immediate decongestive and preload-reducing effects of furosemide on cardiac filling pressures. Although the present study differed from previous experimental models in design and population, dogs with naturally occurring MMVD and CHF exhibited similar short-term reductions in the LA/Ao, LVIDDN, and E peak after diuretic therapy, reflecting comparable decongestive responses under clinical conditions.

The underlying hypothesis of this study was that dogs with advanced and chronic myocardial remodelling would have a limited capacity for structural recovery, as the myocardium and atrial walls may already have lost much of their elastic and contractile reserve (Falk et al. 2013; Hezzell et al. 2014). Based on this assumption, we expected that a greater reduction in chamber size after decongestive therapy would indicate more effective reversal of congestion and be associated with improved outcomes. However, this hypothesis was not supported by the results, as changes in cardiac dimensions during hospitalisation were not related to long-term survival. Similar to observations in human patients with acute decompensated heart failure, in whom most short-term echocardiographic improvements failed to predict long-term outcomes, the reductions in the LA/Ao, LVIDDN, and E wave observed in our study were not associated with survival (Ramasubbu et al. 2012; Deferm et al. 2020). These findings suggest that the short-term echocardiographic response to diuretic therapy should be interpreted primarily as a marker of effective decongestion and stabilisation rather than an indicator of long-term prognosis.

Although baseline echocardiographic indices of remodelling, such as LA/Ao and LVIDDN, were not retained as independent predictors in the final multivariable model, both were significantly associated with survival in the univariable analysis, consistent with prior studies identifying left atrial and left ventricular remodelling indices as key prognostic markers in dogs with MMVD (Borgarelli et al. 2008; Baron Toaldo et al. 2018; Tidholm and Häggström 2022). This finding is consistent with those of previous studies reporting that greater structural remodelling is linked to poorer outcomes in dogs with MMVD (Nakamura et al. 2014; Park et al. 2025). However, because echocardiographic measurements obtained after intravenous diuretic administration were not significantly associated with survival, prognostic evaluation should ideally be based on cardiac dimensions assessed before intensive decongestive therapy. These findings suggest that the degree of remodelling at initial presentation better reflects the chronicity and severity of disease than measurements obtained after stabilisation.

The median duration of hospitalisation in the present study was 3 days, slightly longer than the 2 days reported in dogs and cats hospitalised for acute CHF (Ohad et al. 2018). This difference may reflect variation in discharge criteria and in-hospital management between institutions. At our institution, after marked improvement in respiratory effort at rest, supported by radiographic improvement of pulmonary infiltrates consistent with CHF, patients were typically monitored for approximately 24 hours while receiving the intended post-discharge oral diuretic regimen to confirm sustained clinical stability before discharge.

Dogs in the recurrent group that had received oral diuretics before admission exhibited higher baseline plasma BUN and creatinine concentrations than those in the first-episode group, which is consistent with their more advanced and chronic disease and possible cardiorenal involvement at presentation (Pouchelon et al. 2015; Ronco et al. 2018; Sabetti et al. 2022; Troia et al. 2022; Crosara et al. 2024; Sabetti et al. 2025). In contrast, dogs in the first-episode group showed greater percentage increases in these renal variables during intravenous diuretic therapy, likely reflecting a stronger acute response to initial volume depletion and transient pre-renal azotemia. The recurrent group appeared to have a blunted renal response, which may have been partly attributable to higher baseline values limiting the relative percentage change. The post-treatment LA/Ao also remained higher in the recurrent group, suggesting persistent congestion despite standardised therapy. These dogs also tended to be hospitalised for longer, possibly reflecting a slower stabilisation process in chronic or recurrent CHF. Although baseline renal indices showed only borderline associations with survival in the univariable analyses, these findings together indicate that renal function at admission likely reflects disease chronicity and prior therapeutic adaptation rather than directly influencing long-term outcome.

In the present study, prior oral diuretic therapy before hospitalisation was independently associated with poorer survival. This relationship likely reflects confounding by indication, as dogs that had already received diuretics before presentation were more likely to have experienced recurrent or advanced CHF (Pouchelon et al. 2015; Beaumier et al. 2018). In such patients, chronic medical management may have reduced measured chamber dimensions by lowering preload, thereby masking the actual degree of structural remodelling (de Madron et al. 2011). Consequently, the echocardiographic indices obtained at admission did not retain independent prognostic significance in the multivariable analysis. This observation is clinically intuitive, as dogs already receiving diuretics generally have a more advanced stage of MMVD with shorter expected survival times (Beaumier et al. 2018). Unlike previous studies that evaluated prognosis solely based on echocardiographic parameters, the present findings suggest that in populations including dogs with stage C and D disease, treatment history including the dosage of diuretics should be considered an integral component of prognostic assessment (Vezzosi et al. 2021; Lee et al. 2025).

In the first-episode group (no prior cardiac medication), the MST from the first onset of CHF was 459 days (95% CI 399–519 days). Reported MST after CHF onset in dogs with MMVD varies across studies, with earlier work describing shorter survival (approximately 9 months) (Borgarelli et al. 2008); more recent cohorts have reported longer survival (approximately 15 months) under contemporary management (Romito et al. 2025). Such differences likely reflect variation in case mix, including differences in breed/body size distribution between cohorts, endpoint definitions (including euthanasia practices), and treatment intensity and follow-up. In our cohort, euthanasia was uncommon and generally limited to refractory CHF, which may have influenced observed survival estimates. Because inclusion required paired pre- and post-stabilisation assessments, dogs that died early after admission or could not undergo pretreatment echocardiography were excluded, which may have biased the cohort toward less severely affected dogs and overestimated MST. In addition, management within a 24-hour emergency setting may have facilitated timely reassessment and escalation of therapy.

This study has several limitations that should be acknowledged. As a retrospective analysis, case selection bias and variability in data collection are potential concerns. Only dogs that remained stable enough during hospitalisation to undergo echocardiographic evaluation both before and after diuretic administration were included, which may have resulted in selection bias toward less severely affected dogs. Due to this inclusion criterion, dogs that died shortly after admission or were unable to tolerate pretreatment echocardiography were excluded from analysis, potentially leading to overestimation of the MST. Moreover, treatment protocols, including the dosage, route, and duration of diuretic administration, as well as the timing of post-treatment evaluations were not standardised among cases, which may have influenced the magnitude of changes observed in echocardiographic and biochemical variables. Specifically, the initiation and duration of supplemental oxygen therapy after clinical stabilisation, as well as the timing of post-treatment echocardiographic reassessments, were determined at the attending clinician’s discretion until discharge. This variability in the timing of follow-up echocardiography may have resulted in differences in the interval from treatment initiation and cumulative diuretic exposure at the time of measurement, thereby influencing the magnitude of observed echocardiographic changes. Although echocardiographic measurements were performed by experienced clinicians, interobserver variability cannot be completely excluded and may have influenced certain quantitative parameters such as the LA/Ao or LVIDDN. In addition, because the study population consisted of dogs with acute CHF, echocardiographic data obtained before clinical stabilisation were limited, as detailed assessments of other echocardiographic parameters at the onset of decompensation were not always available. Despite these limitations, this study provides novel clinical insight by characterising the short-term changes before and after diuretic therapy in dogs with naturally occurring MMVD and CHF and by evaluating their relationships with long-term survival.

In dogs with CHF secondary to MMVD, short-term reductions in cardiac dimensions and body weight or transient increases in renal values after intravenous diuretic therapy were not associated with long-term survival. Although indices of cardiac remodelling showed prognostic relevance in the univariable analysis, only prior diuretic therapy remained independently associated with outcome. These findings indicate that in stage C MMVD, prognostic assessment should not rely solely on echocardiographic but must also incorporate treatment history, as previous decongestive therapy reflects disease chronicity and substantially influences survival.

## Supplementary Material

Supplemantary Table.docx Supplemantary Table.docx
