# Supplementary material for: Prognostic relevance of short-term changes in body weight, renal indices, and echocardiographic variables after intravenous diuretic therapy in dogs with myxomatous mitral valve disease hospitalized for pulmonary edema
Source: Vet Q. 2026 Mar 24;46(1):2648250. doi: 10.1080/01652176.2026.2648250 (PMC13015027; doi:10.1080/01652176.2026.2648250)
Supplement: Supplemantary Table.docx [file TVEQ_A_2648250_SM1267.docx]

**Table S1.** **Extra-cardiac comorbidities identified at baseline in 50 dogs with MMVD.**

| Category | n (%) | Specific diseases |
| --- | --- | --- |
| Dogs with ≥1 comorbidity | 30 (60%) | - |
| Urogenital / Urinary / Reproductive | 19 (38%) | Chronic Kidney Disease (n=13; IRIS Stage 1: n=6, 2: n=5, 3: n=2),  Urolithiasis/Renal calculi (n=2), Benign Prostatic Hyperplasia (n=2), Cystitis (n=1), Cystic endometrial hyperplasia (n=1) |
| Hepatobiliary / Hepatic / Splenic | 13 (26%) | GB mucocele/sludge (n=7), Hepatic mass/nodule (n=3), Splenic mass/nodule (n=2), Vacuolar hepatopathy (n=1) |
| Gastrointestinal | 11 (22%) | Gastritis/Enteritis (n=7), Pancreatitis (n=4) |
| Respiratory | 6 (12%) | Tracheal collapse (n=5), Chronic bronchitis (n=1) |
| Endocrine | 4 (8%) | Hyperadrenocorticism (n=3), Hypothyroidism (n=1) |
| Neoplasia | 3 (6%) | Mammary tumor (n=2), Transitional cell carcinoma (n=1) |
| Neurologic | 1 (2%) | Cerebral infarction (n=1) |
| Miscellaneous | 1 (2%) | Glaucoma (n=1) |

Dogs could have more than one extra-cardiac comorbidity; therefore, counts across categories may exceed the number of affected dogs. Extra-cardiac comorbidities were identified and managed according to routine clinical practice. Chronic kidney disease staging was performed according to the IRIS guidelines. Gastroenteritis was recorded as a clinical and/or ultrasonographic presumptive diagnosis. No dogs underwent surgical intervention for extra-cardiac comorbidities during the observation period. The dog with TCC received piroxicam only and did not undergo cytotoxic chemotherapy.

International Renal Interest Society; IRIS
